# Eave tubes for malaria control in Africa: an introduction

**DOI:** 10.1186/s12936-016-1452-x

**Published:** 2016-08-11

**Authors:** Bart G. J. Knols, Marit Farenhorst, Rob Andriessen, Janneke Snetselaar, Remco A. Suer, Anne J. Osinga, Johan M. H. Knols, Johan Deschietere, Kija R. Ng’habi, Issa N. Lyimo, Stella T. Kessy, Valeriana S. Mayagaya, Sergej Sperling, Michael Cordel, Eleanore D. Sternberg, Patrick Hartmann, Ladslaus L. Mnyone, Andreas Rose, Matthew B. Thomas

**Affiliations:** 1 In2Care BV, Marijkeweg 22, 6709 PG Wageningen, The Netherlands; 2 CTF2000, Baaikensstraat 11, 9240 Zele, Belgium; 3 Environmental Health and Ecological Sciences Thematic Group, Ifakara Health Institute, PO Box 53, Ifakara, Tanzania; 4 Biogents AG, Weißenburgstraße 22, 93055 Regensburg, Germany; 5 Center for Infectious Disease Dynamics and Department of Entomology, The Pennsylvania State University, University Park, USA

**Keywords:** Vector control, Resistance, Eave tube, House modification, Africa, Malaria elimination

## Abstract

In spite of massive progress in the control of African malaria since the turn of the century, there is a clear and recognized need for additional tools beyond long-lasting insecticide-treated bed nets (LLINs) and indoor residual spraying (IRS) of insecticides, to progress towards elimination. Moreover, widespread and intensifying insecticide resistance requires alternative control agents and delivery systems to enable development of effective insecticide resistance management strategies. This series of articles presents a novel concept for malaria vector control, the ‘eave tube’, which may fulfil these important criteria. From its conceptualization to laboratory and semi-field testing, to demonstration of potential for implementation, the stepwise development of this new vector control approach is described. These studies suggest eave tubes (which comprise a novel way of delivering insecticides plus screening to make the house more ‘mosquito proof’) could be a viable, cost-effective, and acceptable control tool for endophilic and endophagic anophelines, and possibly other (nuisance) mosquitoes. The approach could be applicable in a wide variety of housing in sub-Saharan Africa, and possibly beyond, for vectors that use the eave as their primary house entry point. The results presented in these articles were generated during an EU-FP7 funded project, the mosquito contamination device (MCD) project, which ran between 2012 and 2015. This was a collaborative project undertaken by vector biologists, product developers, modellers, materials scientists, and entrepreneurs from five different countries.

## Background

Virtually all countries that signed up for the Millennium Development Goals in 2000 have shown dramatic advances in reducing malaria morbidity and mortality (as part of MDG6) over the last 15 years. Global malaria incidence has dropped by an estimated 37 % and mortality by 58 % [1]. Close to one billion insecticide-treated bed nets were distributed in sub-Saharan Africa and have been pinpointed as the primary contributor (68 %) to the observed reduction in *Plasmodium falciparum* prevalence in children 2–10 years of age, which dropped from 33 to 16 % between 2000 and 2015 [2]. Combined with indoor residual spraying, these two vector control interventions made up for 78 % of the estimated 663 million malaria cases averted since the turn of the Millennium. This dramatic and highly encouraging progress has fuelled the belief that global eradication of malaria is feasible, and maps to show how its distribution will shrink to zero by 2040 have been drawn up [3], backed by equally optimistic reports [4].

Considering the fact that vector control has played such a prominent role in these successes, there are two critical issues to consider with regards to its future role in eradication efforts. First, it is now widely accepted that the current two major tools for vector control, LLINs and IRS, both highly effective alone or in combination, will not reduce malaria incidence to zero in high transmission settings [5]. Second, insecticide resistance in the major African malaria vectors, in some countries against several classes of public health insecticides recommended by the World Health Organization, is already widespread and increasing in intensity [6, 7]. Without novel public health insecticides [8] and strategies to manage insecticide resistance [9, 10], it will be difficult to sustain the gains of the last decade [11]. Beyond new actives there is also a dire need for novel delivery tools that can be integrated with current methods, or combined with alternative approaches like larval source management [12] as part of integrated vector management campaigns [13].

Although house improvement for malaria control has a long history [14, 15] and contributed significantly to malaria elimination in Europe and the USA [16], its role in developing countries remains surprisingly small. A recent systematic review and meta-analysis, however, showed that housing is an important risk factor for malaria. It was concluded that, although only one housing intervention study has produced positive clinical outcomes to date [17], further studies on house improvement are warranted [18].

This series of articles introduces a novel house-based malaria vector control intervention called the ‘eave tube’, which combines modifications to make a house more ‘mosquito-proof’ with an innovative way of delivering insecticidal active ingredients. The articles highlight key advances in technology development to date, including initial proof of concept studies, exploration of mode of action, model evaluations, and feasibility for scale-up.

## Rationale

For several years it has been argued that the development of novel tools for anopheline vector control should be guided by a thorough understanding of their ecology and life history behaviours [19]. Current strategies (LLINs and IRS) focus on the fact that in many parts of Africa, during the host-seeking process, female mosquitoes will enter the house at night to gain access to a human host. LLINs target these mosquitoes prior to feeding, IRS afterwards. Both methods have clearly been proven to have public health benefits [20, 21].

The house and the peri-domiciliary domain are closely linked to disease, where up to seventy per cent of infectious disease transmission occurs [22]. This focal transmission results from the shift from nomadic to more sedentary lifestyles and the development of agricultural practices, leading to adaptation of vectors to changed landscapes as well as an increased affinity with human blood. Anthropophagy and utilisation of man-made environments (both indoors and outdoors) thus created an ideal setting for efficient transmission of vector-borne pathogens. It is, therefore, not surprising that the two most commonly used malaria vector control tools in Africa (i.e. LLINs and IRS) are house-based, since mosquitoes are predominantly nocturnal and commonly feed indoors. Although, due to intensified use of LLINs, it has been observed that certain vectors are evading fatal exposure to insecticides indoors by feeding outdoors [23, 24], it has also been determined that outdoor feeding is almost always preceded by attempts to feed indoors [25]. This behaviour indicates that effective house-based killing methods should still result in effective control, even for species in which plasticity in endophagy is observed.

**Fig. 1:**
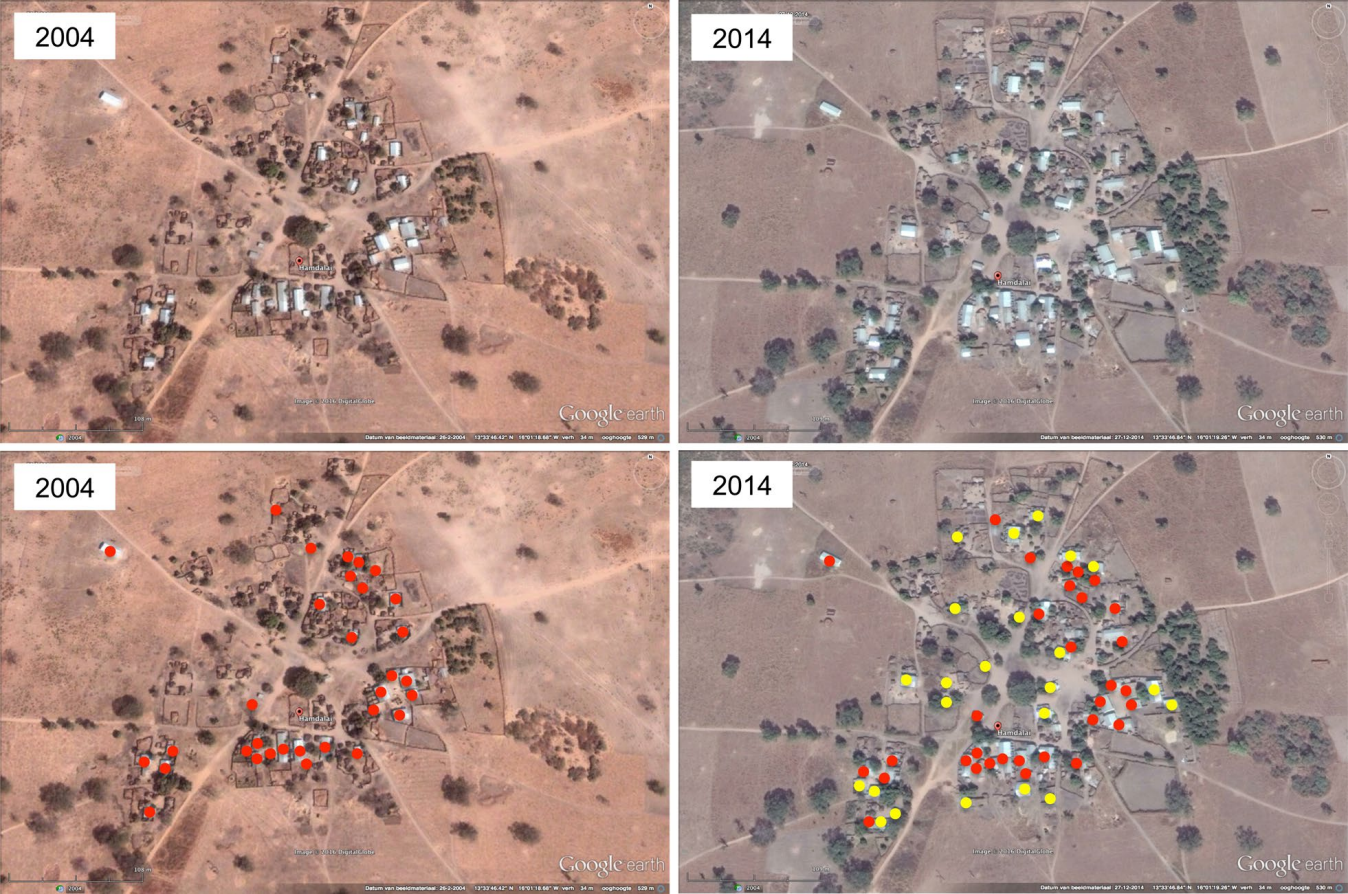
Changes in house design, Hamdalai village, The Gambia. Using Google earth imagery it can clearly be seen that the proportion of houses with corrugated iron sheet roofing in 2004 (*red dots*) has increased over the last decade both within the village and at its periphery (2014; *yellow dots*). This phenomenon is prevalent across Africa

**Fig. 2:**
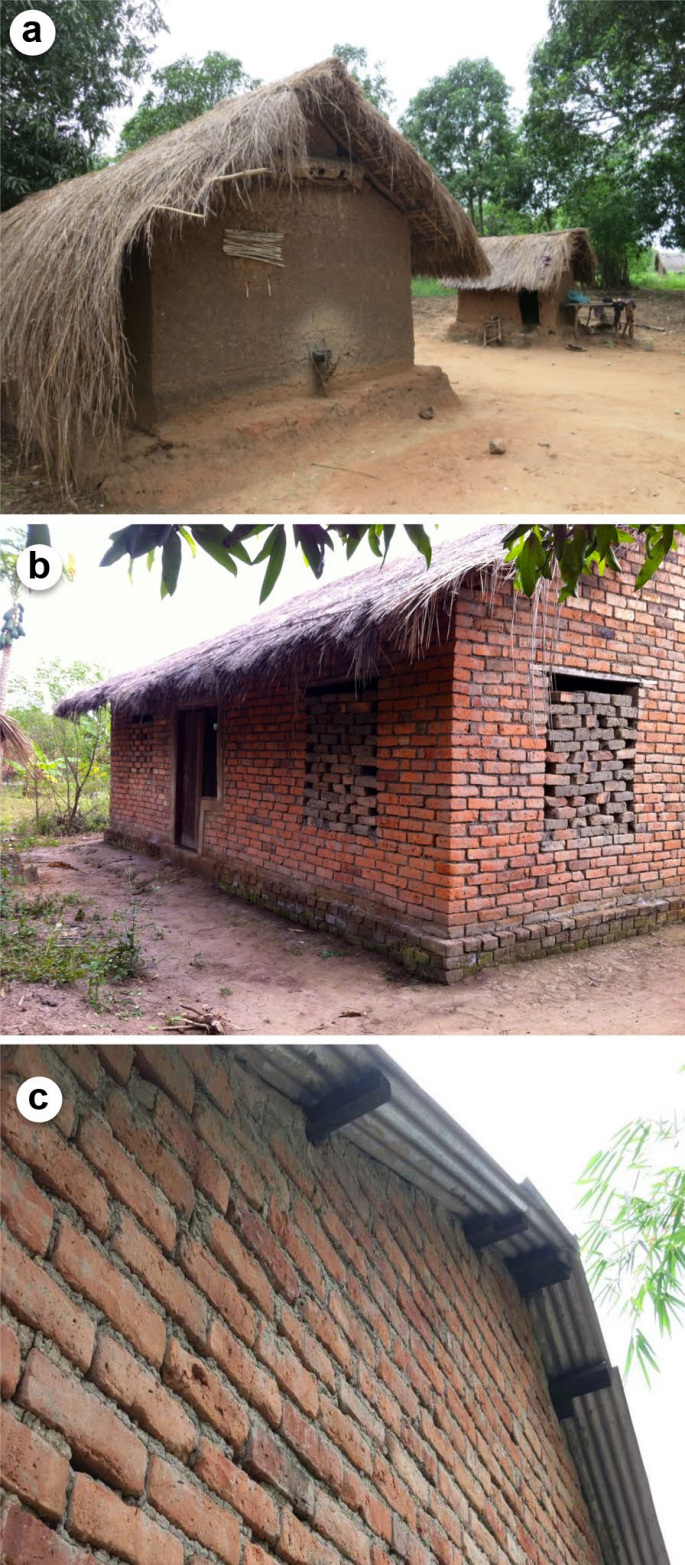
Changes in house design, Kilombero Valley, southern Tanzania. Traditional mud wall and grass thatch houses (**a**) are being replaced by grass thatch and (burnt) brick wall houses (**b**) and subsequently corrugated iron sheet roofing is installed (**c**)

Housing in Africa is currently undergoing design changes at an unprecedented rate. Traditional materials are being replaced with more modern ones that combine features of lower cost and/or durability. Walls that consisted of mud or clay are being replaced with walls consisting of (burnt) bricks or concrete blocks. The same applies to roofing where, across the continent, traditional grass thatch roofs are being replaced with corrugated metal sheet roofing (Fig. 1). Not only are these alternative materials more durable and cheaper over time, they also modify the dynamics of malaria. Given the poikilothermic nature of mosquitoes, changes in indoor climate will affect their survival, blood meal digestion and egg development, as well as parasite development [26, 27]. The dampening effect of a thick grass thatched roof, which keeps the house cooler during the day and warmer at night, is completely lost with metal sheets, resulting in more extreme fluctuations of both temperature and humidity, which combined, influence comfort for house occupants. Moreover, when these house modifications are taking place, many house owners seal the eaves of the house in order to reduce indoor mosquito biting. Apparently, given that it is much more difficult to seal the eaves when the roof is made out of grass thatch, sealing it to prevent mosquito entry is preferred over cooling of the house through air passage through the open eaves. This results in a reduction in indoor comfort, which may result in reduced use of LLINs (these being too hot to sleep under [28]) and an increase in smoke-induced ailments when cooking is done indoors [29]. Besides house modification, large numbers of new houses will be constructed in Africa in the coming decades; estimates indicate some 144 million new structures in rural parts of Africa by 2050 [30]. Africa’s economic growth, with an average expected GDP increase of 6 % per annum over the next decade [30], will result in wealth creation that can be deployed to improve housing so that it becomes less prone to invasion by vectors (Fig. 2).

These alterations in house design present opportunities for the development of novel vector control tools [31]. Eaves, which constitute the gap between the roof and the walls of houses, have long been incriminated as the primary entry point into houses for malaria vectors [32–36], and have formed the basis for malaria control interventions, notably through the use of eave curtains [37, 38] and, more recently, push–pull interventions [39]. Pyrethroid-treated eave curtains have been shown to reduce malaria morbidity and mortality [20, 40], which clearly demonstrates that the eave is an effective place to target host-seeking mosquitoes when they try to enter [41].

## Conceptualization

In late 2012 a diverse group of researchers put together a project called the ‘mosquito contamination device (MCD) project’. The project, supported by the European Union Seventh Framework Programme, aimed to develop a number of novel malaria mosquito control tools. The motivation was to develop technologies that could be made operational in as short a time frame as possible. As such, the project was not structured as a conventional research project and this was reflected in the diverse nature of the project partners, which included vector biologists, product developers, modellers, materials scientists, and entrepreneurs from five different countries.

When rationalizing a novel control tool the following eight criteria were considered essential:

1. Human contact with insecticides should be minimized;
2. Application of novel insecticides (including resistance-breaking actives like entomopathogenic fungi) beyond the currently approved ones should be feasible; possibly even combinations of different classes of insecticides should be considered;
3. The amount of insecticide applied per house should be reduced as much as possible to save costs and reduce possible impact on the environment and non-target organisms;
4. The approach should not compromise indoor comfort of house occupants or have any other health impacts;
5. The technology should preferably operate passively without any active engagement of house occupants;
6. The approach should become widely accepted and adopted in affected communities and possibly form part of income-generating activities;
7. The tool should be cost-competitive with LLINs and/or IRS, easy to mass-produce, and easy to install and maintain/service;
8. The technology should be able to operate without depending on electricity or addition of mosquito attractants, notably carbon dioxide.

During the MCD project-planning workshop that was held in Ifakara, Tanzania, in February 2013, the authors visited several villages in the Kilombero Valley and took note of the changes in house design. The observation that house owners were regularly closing the eaves when they installed corrugated metal sheet roofing triggered the idea for eave tubes for three primary reasons. First, the closure of the eaves by house owners resulted in the obstruction of airflow and thus possible changes to indoor comfort. Partial opening of the eaves (through the installation of tubes) would potentially reinstate the airflow and thus could improve comfort. Second, by installing tubes and reinstating airflow, anopheline mosquitoes would be able to perform their natural behaviour whilst host-seeking, i.e. they would respond to host odours [42] emanating from the tubes (at eave height) and fly into these. By installing a physical barrier inside the tube (i.e. netting), mosquitoes could be prevented from entering the house and at the same time be exposed to insecticidal agents that were applied to the netting. In doing so, mosquitoes would not simply be presented with a mosquito-proof house and be diverted to other houses in the vicinity but have a significant chance of becoming exposed to insecticide during any of the 3–4 host-feeding cycles before becoming infectious and contribute to malaria transmission. The third reason combined several possible advantages in that (a) it would be possible to use bioactivities in a safe place beyond the reach of children and other house occupants, (b) provide an opportunity to use novel (bio) pesticides or combinations thereof, and (c) use much less insecticide, so creating opportunities for utilizing products that might be cost prohibitive in conventional applications such as IRS. A secondary reason for focusing on tubes was the reduced requirements for netting compared to eave curtains, and since one of the partners (In2Care BV) had developed a novel electrostatic coating for application on netting that is pyrethroid-resistance breaking [43], the authors wanted to use only minimal quantities of this special netting for treatment with powder formulations of insecticides.

**Fig. 3:**
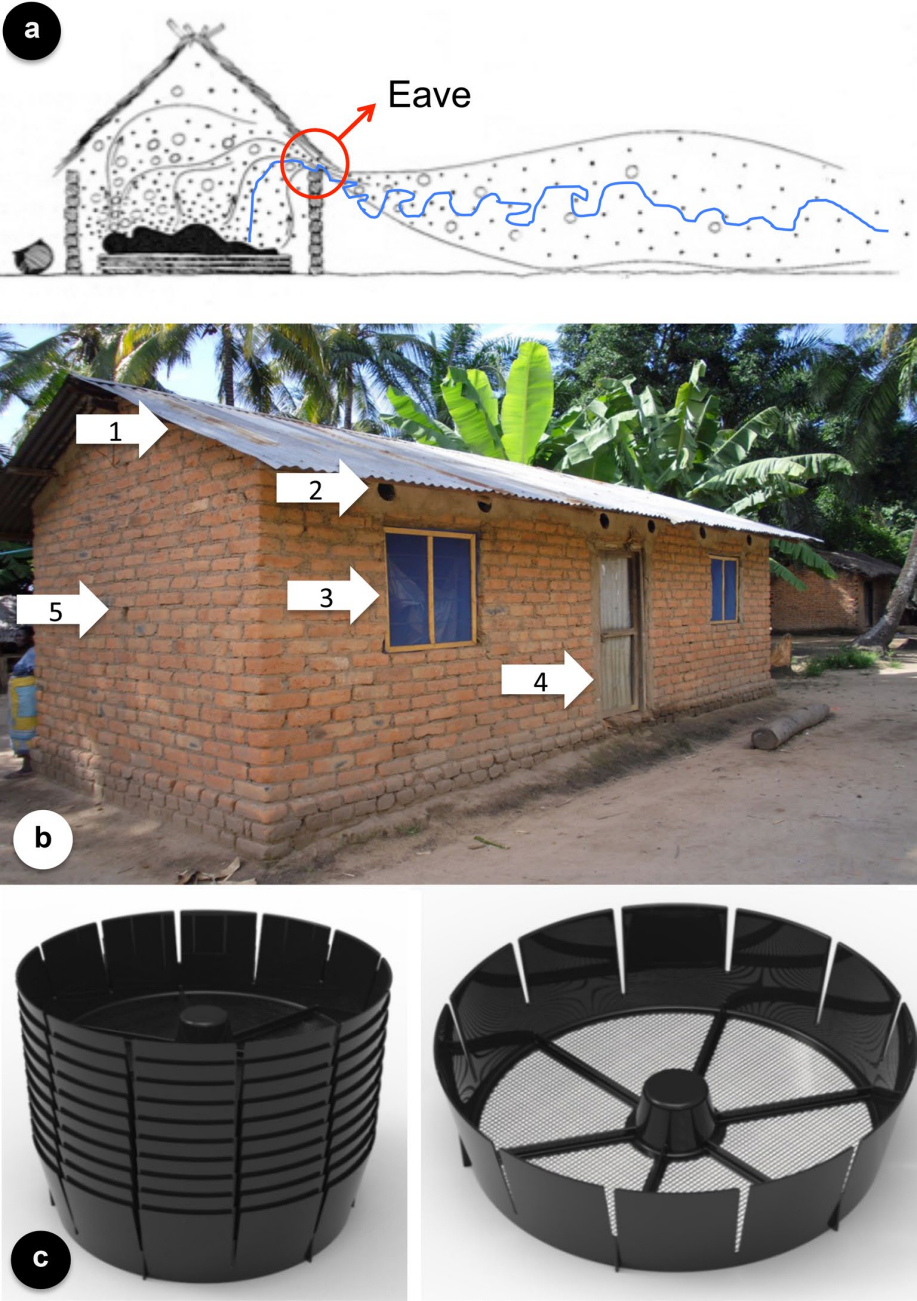
The eave tube concept. **a** African anophelines fly upwind in host odour plumes (*blue line*) and enter houses through the gap between the roof and the walls, the eave (*red circle*); drawing modified after [44]. **b** A house in southern Tanzania fitted with eave tubes and rendered mosquito-proof through fitting of window screening and sealing of the eaves. The house modification comprises: *1* Closing the eaves, *2* Installation of eave tubes, *3* Fitting of window screening, *4* Repairs of the door (where necessary), and *5* Closing of cracks and holes. **c** Eave tube inserts (stack on the left), fitted with insecticide-treated netting that fit inside PVC pipes. Development of this insert is described in Snetselaar et al. [pers. comm.]

Eave tube technology does not only consist of eave tubes but also window screening (with untreated netting) as well as sealing of cracks and gaps in walls and (whenever possible) improvement of the door (Fig. 3). These are commonly used strategies to keep mosquitoes out of the house and will not be reported on separately here. However, whenever mention of ‘eave tubes’ is made, it should be clear that this represents a suite of modifications, i.e. closure of the eaves, installation of the tubes with insecticide-treated netting, rendering all windows mosquito proof through the installation of (untreated) window screening, door repairs to reduce the possibility for mosquito entry, and general closure of openings and cracks, with the same goal.

## Research and development

In the research and development phase that followed the initial conceptualisation, research focused primarily on semi-field studies that facilitated quick evaluations of key characteristics of the technology. These studies were undertaken both in Tanzania (at the Ifakara Health Institute in Ifakara; [Sternberg et al. pers. comm.]) and at the Thomas Odhiambo campus of ICIPE at Mbita Point, Kenya [Snetselaar et al. pers. comm.], so that different strains and anopheline species could be tested. The data generated from these studies formed the basis for a parameterized modelling exercise to gain further insight on the potential of the technology in terms of reducing transmission alone or in combination with LLINs or IRS [Waite et al. pers. comm.]. Both semi-field and field studies were undertaken to study the behaviour of mosquitoes when entering the eave tubes. Videographic studies were conducted to quantify behaviours inside the eave tubes and measure exposure durations upon contact with different active ingredients [Sperling et al. pers. comm.]. Finally, studies were undertaken in the village of Mngeta (Tanzania) to assess community acceptance of eave tube technology and undertake an operational feasibility study that incorporated some 1900 houses. This enabled us also to gain insight in the economics of this approach when compared with LLINs or IRS [Knols et al. pers. comm.].

This series of six articles provides an overview of the research that was undertaken over the past 3 years that has led up to a Phase III randomized controlled trial that is currently being prepared in Côte d’Ivoire.

## Abbreviations

IRS: indoor residual spraying
LLIN: long-lasting insecticide-treated net
MCD: mosquito contamination device
MDG: Millennium Development Goal
